# Imagining an ethics for synthetic biology

**DOI:** 10.3389/fgene.2026.1746379

**Published:** 2026-02-13

**Authors:** Varsha Aravind Paleri, Kristien Hens

**Affiliations:** Department of Philosophy, Centre for Ethics, Universiteit Antwerpen–Stadscampus, Antwerpen, Belgium

**Keywords:** bioethics, embedded ethics, non-dualism, slow science, synthetic biology, non-western, ethics-by-design

## Introduction

Humanity is facing many complex challenges. It is therefore self-evident that many endeavours in biotechnology are framed in terms of offering promising solutions for these challenges. Synthetic biology is an umbrella term for technologies that aim at designing and constructing new biological parts, devices, and systems, as well as the redesign of existing natural biological systems or the creation of complex artificial biological systems (Anderson et al., 2012; Bedau et al., 2009; Saukshmya and Chugh, 2010). Possible applications include medicine and environmental applications such as bioremediation. For example, in biomedicine, synthetic biology applications can accelerate molecular production and facilitate diagnosis through different health-monitoring systems using biochips to detect physiological changes (Yan et al., 2023). In agriculture, synthetic biology can aid nitrogen fixation, reduce the use of synthetic fertilizers, improve the nutritional value of plants, aid in soil remediation, and change the production mode of chemical pesticides to biopesticides (Ye et al., 2024). At the same time, several concerns have been raised regarding biosafety and biosecurity, and the dangers of such technologies being used in terrorist attacks. Beyond such application-related dangers, such technologies may invoke a more fundamental fear, one that is reminiscent of the lack of acceptance of GMO-modified foods in Europe (Greens/EFA, 2025; Bonny, 2003). We treat the GMO debate as a historical analogue to synthetic biology and not as an equivalent field, although the issues discussed recur across biotechnology more broadly. Some scholars consider it intrinsically wrong and dangerous to modify life, whereas others argue that it is exactly humanity’s task to do so (Aravind Paleri and Hens, 2023). Justice is another concern, one that has been raised with regard to GMOs by thinkers such as Vandana Shiva, and that may apply to synthetic biology applications as well (Pathak and Subudhi, 2019). Whose lives and livelihoods will be negatively affected by these technologies if they remain in the hands of private companies, and who will benefit? It seems that ethical reflection is needed.

This comment is the result of a four-year research project on the ethics of Synthetic Biology. Both from our review of the existing ethical literature and from our engagement with scientists in synthetic biology labs, we found a lack of scientific voices and limited engagement among scientists themselves in discussions of ethical issues in Synthetic Biology. Moreover, some of the ethical concerns mentioned were framed in dichotomies, such as nature vs. technology or human vs. more-than-human life (Aravind Paleri and Hens, 2023; Paleri, 2025). In this comment, we argue that for an ethics of Synthetic Biology to be able to adequately answer ethical issues and to ensure that neither the risks nor the benefits of this technology are downplayed, at least three issues need to be addressed, beyond what is currently discussed in the literature. First, we argue that, by reflecting on the moral status of synthetically created organisms, an engagement with non-Western ontologies can provide an alternative perspective on current ethical concerns. Second, we point out that the ‘ethics of Synthetic Biology, and perhaps of biotechnology and science in general, should not be conceived of as an afterthought. Rather, ethical reflection, as well as the engagement of the lived experiences and opinions of all stakeholders from the outset, should be part of any project from its inception. Third, we argue for an extensive mapping of those who will be affected by the techniques at hand, and an engagement of the lived experiences and opinions of all stakeholders, from the start.

## Engaging with other worldviews

First, in our review of the existing literature, we have found that in many ethical discussions surrounding synthetic biology, the acceptability of technologies hinges on whether we are actually creating life or dealing with machines (Aravind Paleri and Hens, 2023). In some of the papers we analysed, it is often assumed that ‘natural’ is better than artificial, and life has a sanctity that non-life does not (Bedau et al., 2009; Braun et al., 2013; Heyd, 2012). While doing the literature review, it struck us first that ontological-ethical issues relating to ‘creating life’ and ‘playing God’ were more prevalent than others, such as the societal and economic impact of synthetic biology (Christiansen, 2016; Kaebnick et al., 2014; Bedau et al., 2009). Moreover, starting from dichotomies rooted in Western traditions, such as life versus non-life and biology versus machine, we felt, may limit moral imagination by isolating human agency from the ecological and spiritual continuities that sustain life. Engaging with worldviews that do not take such dichotomies for granted may help us see technology through a different light. To illustrate, we provide the example of Hinduism. As a Hindu, one of us was acquainted with a worldview that views life as an interconnected continuum rather than a hierarchy of beings. In this view, humans are not external creators “playing God” but co-participants in the ongoing unfolding of life (*prakṛti*), where every entity, living and non-living, possesses relational value (Baindur, 2015). Taking such a non-dualistic ontology seriously may help reframe the core questions that have haunted synthetic biology ethics from the start and nuance the debate beyond taboo or unconditional acceptance. Indeed, from a Hindu perspective, the creation of synthetic entities is not an act of domination over nature but a modification within a shared field of existence, calling for humility and awareness rather than control. Ethical reflection thus moves from abstract principle-based judgment to *attunement*, a situated practice of recognizing one’s interdependence with material, biological, and spiritual processes. Concepts such as *Dharma* (context-specific right action), *karma* (consequences), *Ñāna* (Knowledge), *Vairāgya* (Detachment), and *Ahimsā* (non-harm) provide normative guidance grounded in relational responsibility rather than categorical rules (Paleri, 2025). For example, engaging with such approaches can be a starting point for conversations about the role of more-than-human life as active partners in the synthetic biology labs. We argue that by engaging with different worldviews and views on life and non-life, ethical deliberation in synthetic biology can engage diverse ontologies and epistemologies and evolve beyond age-old Western discussions centering around the question of whether we are allowed to ‘play God’. Such pluralism can thus enrich responsible innovation by acknowledging multiple ways of knowing and valuing both living and non-living things. Moreover, it may help us focus more on the other important aspects of scientific development, such as the social and justice issues, than on ethical questions hinging on questionable ontological distinctions.

## Ethics from the start

Second, in biotechnology, ethics is often treated as an afterthought, limited to ethical dossiers and approval committees. On the one hand, scientists do not take ethics too seriously, and on the other hand, they are fearful of strict regulations and bans. This is a missed opportunity for ethics and science to co-create valuable outcomes from the onset. Several scholars in STS have paved the way in embedded ethics approaches (Buedo et al., 2024; Lunshof and Rijssenbeek, 2024; Reijers et al., 2018; Kenis et al., 2025). For example, Lunshof and Rijssenbeek propose a form of collaborative ethics as a model in which ethicists and philosophers work in real time alongside life scientists throughout the research process, rather than limiting ethics to pre-approval committee reports or *post hoc* review reports. Our own experience in a large interdisciplinary synthetic biology consortium, POSSIBL, is similar to theirs. We set out to embed ethics from the very beginning and carried through every stage of the research process. However, this is not an easy task, as we have documented in our Bioethics-in-Science paper (Kenis et al., forthcoming). The first step to achieve this is to bring a change in the relationship between ethicists and scientists. Ethicists must be seen as collaborators within the project and not solely as mere consultants. The relationship between scientists and ethicists needs to be fostered, and this co-development should occur as the research progresses. Ethics cannot thrive through occasional check-ins or after-the-fact reviews. From our personal experience in the project, we realized that trust and meaningful ethical reflection were not possible only through formal meetings. Rather, what helped us gain this trust and enrich the ethical interaction was more through everyday interactions: informal conversations over lunch, in the corridor, leading to moments of shared problem-solving or brainstorming sessions. While the continuous presence of an ethicist in the lab was critical for such interactions, we hit a constraint here. Being a single ethicist in a big consortium of synthetic biology labs meant we could not be present all the time in all labs, which restricted the intensity of the interaction with the researchers. At the same time, dedicating one ethicist per lab can also seem unlikely under current funding models. Nonetheless, for ethics to truly engage with science, it should extend beyond formal interventions such as getting approval from ethics committees and should include learning from the everyday experiences of researchers (Jongsma and Bredenoord, 2020; Lunshof and Rijssenbeek, 2024; Reijers et al., 2018). From our experience and learnings of this project, we created an SOP that can act as a guide for early-stage ethicists setting out to integrate ethics in science projects (Paleri and Hens, 2025).

## Reaching out, slowing down

Third, beyond ethics-in-the-lab, ethicists and scientists must also reach out. Indeed, synthetic biology does not develop in isolation. Its outcomes, whether they are beneficial or harmful, potentially reverberate far beyond the laboratory. In her book *Another Science is Possible: A Manifesto for Slow Science,* Isabelle Stengers argues that GMOs reveal how research locked into a high-speed, benchmarked logic tends to ignore complex “matters of concern”, such as large-scale field deployments, issues of biodiversity, patenting, and the transformation of whole agricultural systems in favour of laboratory-defined “matters of fact” (Stengers, 2018). The same risks apply to Synthetic Biology. Synthetic Biology is not being developed only in isolation, and its effects, positive or negative, will inevitably be carried by people and the environment far beyond where it originates. Engaging with varied worldviews and epistemologies enables us to approach complex global challenges with greater nuance (Hoelting et al., 2024; Stein et al., 2024). The inclusion of diverse knowledge systems and considerations of lived experiences broadens the ethical landscape and provides a more holistic foundation for assessing the implications of emerging biotechnologies. These perspectives often offer context-specific insights and locally grounded strategies that may not be visible through dominant paradigms. Working alongside, rather than ignoring, these perspectives ensures that technological development is attuned to a wider range of needs and values (Ijatuyi et al., 2025; Orlove et al., 2023). Isabelle Stengers reminds us that technoscience risks “becoming deaf to what it cannot translate,” and that genuine progress depends on cultivating the capacity to be affected by other forms of thought and practice (Stengers, 2018, p.63). In the context of Synthetic Biology, this means working with rather than on others, such as scientists, ethicists, communities, microbes, and ecosystems. Concretely, projects in Synthetic Biology should ask themselves, who is affected by my research, and how do we make sure that their viewpoints and experiences are taken into account. It is especially here that embedded approaches and ethicists-in-the-lab can help achieve such slow science. Although ethicists may only have a limited impact on some of the causes of ‘fast science’, such as the way projects are financed, having ethicists in the lab, either physically at regular periods, attending team meetings, and engaging with other scientists, may be one step forward towards slowing down science. By slowing down, we also create the time to think not only about the hidden and invisible labour of people, but also about the long-term consequences of our current scientific decisions. This pause can help us reflect on how our innovations and decisions may unintentionally redistribute benefits and harms across individuals and communities. A well-documented example is the development of semi-synthetic artemisinin, which, being an important development of synthetic biology, ended up disrupting the livelihoods of farmers who depended on cultivating Artemisia annua, revealing how unknown and unforeseeable ethical impacts can extend far beyond the laboratory (Case Study, 2014). Slow science helps us pause, reflect, and ask questions about the innumerable contributions made by not only humans but also invisible contributors, like the more-than-human entities. The Labour Provenance framework is one such initiative that helps uncover the usually invisible contributions of human and more-than-human entities who are visibly and invisibly implicated in the research. This framework argues for acknowledging and accounting for these collaborators rather than merely treating them as resources (Chen et al., 2025). Similar to this framework, during one of our lab visits, we collaborated with artists who often work with Synthetic biology labs, and we created a workshop that focused on the more-than-human life actively contributing to our daily research. Engaging scientists and fellow ethicists in different worldviews and making them see things differently is not an easy task. In this workshop, we, along with the artists, employed an art-based approach, incorporating visual storytelling and hands-on exercises with fungi, to invite participants to reimagine their relationship with the microorganisms they were working alongside. These interactions also sparked discussions on the responsibilities scientists have not only to the human world, but the world beyond, comprising more-than-human life and the ecosystem we share (Revistas, 2026).

## Conclusion

In sum, an ethics of Synthetic Biology should be one that listens, slows down, and learns from the different actors and worlds it touches. In order to nourish such ethics, an engagement with different non-Western ontologies is fruitful to go beyond stale discussions hinging on problematic dualisms. Following Stengers’ call for a “slow science,” we must cultivate scientific practices that are able to hear what dominant paradigms tend to silence. Embedding ethics from the start, working in genuine collaboration across disciplines and communities, and acknowledging the lived experiences and worldviews of those affected are the conditions of possibility of technological advancement, not hurdles to be taken.
